# Caffeine as a Modulator in Oncology: Mechanisms of Action and Potential for Adjuvant Therapy

**DOI:** 10.3390/ijms26136252

**Published:** 2025-06-28

**Authors:** Nina Rembiałkowska, Alina Demiy, Alicja Dąbrowska, Jakub Mastalerz, Wojciech Szlasa

**Affiliations:** 1 Department of Molecular and Cellular Biology, Faculty of Pharmacy, Wroclaw Medical University, Borowska 211A, 50-556 Wroclaw, Poland; wojciech.szlasa@umw.edu.pl; 2 Faculty of Pharmacy, Wroclaw Medical University, 50-556 Wroclaw, Poland; 3 Faculty of Medicine, Wroclaw Medical University, Pasteura 1, 50-367 Wroclaw, Polandjakub.mastalerz@student.umw.edu.pl (J.M.); 4 Lower Silesia Centre for Oncology, Pulmonology and Hematology in Wroclaw, 53-439 Wroclaw, Poland

**Keywords:** caffeine, neuroprotection and neurodegenerative, adjuvant therapy, cognitive effects, fatigue management

## Abstract

Caffeine, one of the most widely consumed bioactive compounds worldwide, is gaining recognition for its potential anticancer properties beyond its well-known neurological and metabolic effects. Mechanistically, caffeine exerts anti-tumor activity by modulating key cellular pathways involved in carcinogenesis, including the inhibition of phosphodiesterases, antagonism of adenosine A2A receptors, and disruption of the DNA damage response through ATR-Chk1 pathway inhibition. These actions collectively promote apoptosis, suppress tumor cell proliferation, and impair metastatic spread. In vitro and in vivo studies have demonstrated that caffeine can enhance the cytotoxic effects of chemotherapeutic agents and radiation therapy, suggesting a synergistic role in conventional cancer treatments. Epidemiological data further supports an inverse association between habitual caffeine consumption and the incidence of several cancers, notably liver, colorectal, breast, and prostate cancers. Among these, the most consistent experimental and clinical evidence exists for liver and colorectal cancer, where caffeine’s modulatory effects on inflammation and cell proliferation have been repeatedly observed. Additionally, caffeine’s anti-oxidant and anti-inflammatory properties may contribute to a microenvironment less conducive to tumor initiation and progression. While promising, the anticancer effects of caffeine are influenced by factors such as dosage, individual genetic variability, and cancer type, underscoring the need for further clinical investigation. This review explores the emerging role of caffeine as a potential chemopreventive and adjuvant therapeutic agent in oncology.

## 1. Introduction

Caffeine (1,3,7-trimethylxanthine) (CAF) ranks among the most prevalently consumed psychoactive compounds worldwide, found not only in coffee and tea but also in cocoa, chocolate, soft drinks, energy beverages, and even incorporated into certain analgesic and anti-inflammatory medications. Because of this ubiquitous presence and its pharmacological activity, CAF is often regarded as the quintessential xenobiotic in the human environment [1,2,3]. Studies across in vitro, animal, and epidemiological settings have documented a broad spectrum of CAF biological effects, spanning beneficial anti-oxidant and neurostimulatory actions to potential adverse outcomes when misused or overconsumed. Beyond its classic role as a central nervous system stimulant, CAF exerts complex biochemical influences—acting as an anti-oxidant under some conditions while paradoxically promoting oxidative stress in others [4]—and modulates neuroendocrine pathways via adenosine receptor antagonism and stress hormone release, with downstream implications for immune function and inflammation [5,6]. These immunomodulatory effects raise important questions about CAF’s potential to enhance or inhibit the efficacy of cancer immunotherapies. Moreover, significant inter-individual differences in response—driven by genetic variants in CYP1A2 and adenosine receptor genes (e.g., ADORA2A)—underscore the importance of personalized considerations. This review synthesizes current evidence on CAF dual anti-oxidant/pro-oxidant roles in cancer cell biology, its neuroendocrine–immune interactions relevant to oncology and immunotherapy, and the impact of genetic polymorphisms on these processes. The latter section examines clinical and experimental findings on CAF psychostimulant and neuropsychiatric effects, its influence on neurodegenerative diseases and cognitive aging, and the systemic consequences of long-term consumption. Although widely recognized for its neurological and metabolic effects, caffeine’s relevance in oncology primarily derives from its modulation of cellular redox status, cell cycle checkpoints, and immune regulation within tumor microenvironments. At therapeutically achievable concentrations, caffeine can act as a sensitizer to conventional chemotherapy and radiotherapy by shifting cancer cells toward apoptosis through heightened oxidative stress, inhibition of DNA damage repair mechanisms, and reduced cancer cell proliferation.

## 2. General Mechanisms of Caffeine Action

CAF significantly impacts the body, particularly the nervous system and metabolism, by increasing alertness, enhancing physical performance, and accelerating metabolism. It is often found in combination with polyphenols, as in coffee and tea, which can amplify its effects due to the anti-oxidant properties of polyphenols, providing beneficial effects on the heart and brain. Despite its numerous benefits, excessive CAF consumption can lead to health issues such as sleep disturbances, anxiety, hypertension, and gastrointestinal discomfort. It is essential to adjust CAF intake to individual tolerance levels. CAF acts through several mechanisms, some of which are dose-dependent. Its primary mechanism is the antagonism of adenosine A1 and A2A receptors, which increases central nervous system activity. Blocking these receptors leads to the release of neurotransmitters, such as norepinephrine, acetylcholine, and dopamine, resulting in stimulation, an accelerated heart rate, and increased concentration. CAF also inhibits phosphodiesterase, elevating cell cAMP levels, which causes smooth muscle relaxation and enhanced lipolysis. Activation of A2A receptors in dopamine-rich brain areas boosts dopamine activity, explaining behavioral changes following CAF consumption [7].

**Figure 1:**
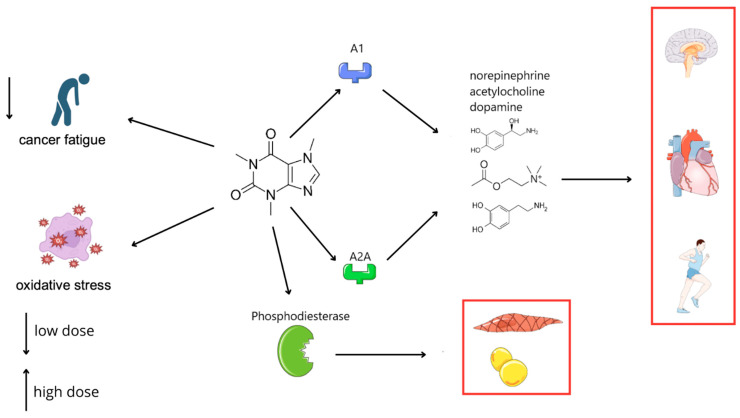
Impact of caffeine on the nervous, cardiovascular, and metabolic systems via modulation of adenosine receptors and phosphodiesterase.

Caffeine’s modulation of the ATR-Chk1 signaling pathway represents a critical mechanism through which it enhances the efficacy of DNA-damaging therapies. ATR-Chk1 is central to the cellular DNA damage response, primarily through cell cycle checkpoint activation. By inhibiting ATR kinase activity, caffeine disrupts checkpoint control, forcing cancer cells harboring unrepaired DNA damage prematurely into mitosis—a process leading to mitotic catastrophe and apoptosis. Preclinical studies have documented caffeine-induced sensitization of cancer cells to chemotherapeutics such as cisplatin, doxorubicin, and camptothecin, as caffeine prevents tumor cells from halting their cell cycle to repair DNA damage. Thus, caffeine-mediated ATR-Chk1 inhibition not only potentiates conventional cytotoxic therapies but also provides a promising rationale for combination treatment strategies aimed at overcoming therapeutic resistance [8,9,10,11]. Figure 1 provides an overview of the biological effects of caffeine (CAF) across various domains discussed in this review.

## 3. Genetic Polymorphisms and Individual Variability in Caffeine’s Effects

Genetic differences play a significant role in how CAF is metabolized and how it affects both oxidative stress and immune function. The enzyme cytochrome P450 1A2 (CYP1A2) is responsible for metabolizing ~95% of ingested CAF in the liver. A well-studied polymorphism in the CYP1A2 gene (−163C>A, rs762551) stratifies individuals into “fast” and “slow” CAF metabolizers. Carriers of the C variant (−163 C) have lower enzyme inducibility and activity, meaning they clear CAF more slowly, whereas the A/A genotype is associated with higher CYP1A2 activity (fast metabolism). These genetic differences can modulate CAF’s impact on both cancer-related oxidative stress and immune–endocrine interactions [12].

In terms of cancer risk and oxidative effects, slower CAF metabolism can prolong CAF presence and enhance its biological effects—for better or worse. An illustrative human study focused on women with BRCA1 mutations (a high-risk group for breast cancer) found that the protective association between coffee intake and breast cancer incidence depended on CYP1A2 genotype. Among women carrying at least one slow-metabolism allele (C allele), regular coffee consumption was linked to a striking 64% reduction in breast cancer risk compared to non-consumers. In contrast, women with the fast metabolizer genotype (A/A) showed no significant risk reduction from coffee. This suggests that a longer exposure to CAF (as occurs in slow metabolizers) may be required to confer cancer-protective, possibly anti-oxidant effects. CAF influence on endogenous anti-oxidant levels might also differ by genotype: as noted earlier, CAF can affect uric acid production via xanthine oxidase, and genetic variability in both CYP1A2 and XO enzymes leads to different outcomes in oxidative stress markers [1]. One review pointed out that inconsistent findings on whether coffee raises or lowers uric acid could be explained by CYP1A2 polymorphisms—individuals with slower CAF clearance may accumulate more pro-oxidant metabolites like uric acid intracellularly, whereas fast metabolizers do not. Similarly, differences in CYP1A2 activity might alter the balance of CAF anti-oxidant versus pro-oxidant action in tissues: a slow metabolizer might experience sustained activation of pathways like SIRT3/SOD2 (extended anti-oxidant effect) and prolonged inhibition of DNA repair checkpoints (extended pro-oxidant stress on cancer cells). By contrast, a fast metabolizer sees CAF peak and clears quickly, potentially blunting both the beneficial and deleterious extremes. In rodents, where genetic knockout or inhibition of Cyp1a2 can mimic slow metabolism, CAF biochemical effects (like increased blood pressure or ROS generation) are often amplified, reinforcing that the metabolism rate is a key modifier [13].

Genetic polymorphisms in CAF molecular targets also modulate their effects. Variants in the adenosine A2A receptor gene (ADORA2A) are known to influence sensitivity to CAF central effects (such as anxiety and sleep disturbance) and are now recognized to affect its immunological and endocrine outcomes as well. A common SNP in ADORA2A (c.1083C>T, rs5751876) has been studied in relation to CAF responses. Individuals with the TT genotype (homozygous for the T variant) are generally more sensitive to CAF; interestingly, this genotype has been associated with greater anti-inflammatory and hormonal responses to CAF in at least one study [14]. In a placebo-controlled trial, researchers found that after a dose of CAF prior to exercise, TT genotype males had significantly larger increases in serum epinephrine, testosterone, and growth hormone compared to C-allele carriers. The TT group also exhibited more pronounced reductions in markers of inflammation post-exercise, consistent with a stronger engagement of CAF adenosine-blocking effects. These findings align with prior observations that TT individuals may derive a larger ergogenic benefit from CAF and show blunted anxiety (or rather, some studies find TT individuals have more anxiety—the data are complex, but clearly the genotype alters receptor function). From an immunological perspective, if the TT genotype confers a heightened A2A blockade by CAF, one could speculate that TT carriers might experience a greater boost in anti-tumor immune activity from CAF. Conversely, those with the CC genotype (adenosine receptor more typical) might not see as much immune benefit and might predominantly experience CAF peripheral effects like jitters or blood pressure rise. While direct evidence in cancer patients is lacking, it is conceivable that ADORA2A polymorphisms influence how well CAF can enhance Th1 cytokine production or T-cell infiltration in tumors.

Apart from CYP1A2 and ADORA2A, other genetic factors can influence CAF’s role in cancer and immunity. Polymorphisms in NAT2 (another enzyme involved in metabolizing coffee components) have been linked to different cancer risks with coffee intake, though NAT2 is less crucial for CAF itself. Genetic variability in anti-oxidant enzymes (such as GSTs or SOD2) might also modulate how CAF pro-/anti-oxidant effects translate into cellular outcomes. For instance, a variant that lowers SOD2 activity could make a person’s cells more susceptible to CAF-induced ROS damage at high doses. Additionally, adenosine A1 receptor variants or downstream signaling protein polymorphisms could alter the neuroendocrine response to CAF (impacting how much cortisol is released, for example). A holistic analysis by Ruiz and colleagues suggested that gender and racial differences in CAF metabolism—partly genetic in origin—correlate with differences in oxidative stress markers and could even affect clinical outcomes. The emerging picture is that pharmacogenomics matters: CAF’s influence on cancer biology is not one-size-fits-all. For instance, a “slow metabolizer” with a tumor might benefit more from CAF’s direct tumoricidal effects (but also needs to watch out for side effects), whereas a “fast metabolizer” may see little effect unless the dose is adjusted. Likewise, someone with a favorable ADORA2A genotype might experience immune stimulation from CAF that complements immunotherapy, while another person might require higher CAF intake to achieve a similar A2A-blocking immunological effect. Recognizing these genetic differences is important for interpreting the mixed results across studies and could guide personalized dietary advice for cancer patients in the future. Researchers are now integrating genetic data into studies of diet and cancer; for example, incorporating CYP1A2 genotype strengthened the observed association of coffee with improved survival in colorectal cancer patients [15]. Such findings underscore that what holds true “on average” may conceal substantial inter-individual variation.

## 4. Caffeine in Cancer

CAF’s impact on the nervous and endocrine systems—notably through blocking adenosine receptors and triggering stress hormone release—has downstream effects on immune surveillance and inflammation [5,6]. These immune effects raise questions about whether CAF might synergize with or antagonize cancer immunotherapies that rely on robust immune activation. Complicating matters, individuals vary in their response to CAF due to genetic polymorphisms in key genes like CYP1A2, which governs CAF metabolism, and adenosine receptor genes (ADORA2A). Hypercalcemia of malignancy occurs in over 40% of cancer patients and is driven primarily by tumor-derived parathyroid hormone-related peptide, illustrating how systemic metabolic derangements can accompany and complicate oncologic management [16].

CAF has demonstrated dual effects on oxidative stress, acting as an anti-oxidant in some contexts while promoting reactive oxygen species formation in others. The net effect often depends on dosage and cellular environment. The establishment of liver metastases depends critically on a permissive microenvironment—characterized by extracellular matrix remodeling, angiogenesis, and inflammatory cytokine signaling—which may be modulated by agents such as CAF in adjuvant settings [17].

Anti-oxidant properties: At physiological or low micromolar concentrations, CAF can scavenge free radicals and bolster cells’ anti-oxidant defenses. For instance, CAF is reported to activate sirtuin-3 (SIRT3) in mitochondria, which in turn enhances the activity of superoxide dismutase 2 (SOD2) by preventing its acetylation. By upregulating such anti-oxidant enzymes, CAF can reduce intracellular ROS and protect cells from oxidative DNA damage. In normal tissues, this anti-oxidant action is largely beneficial—it may contribute to the observed epidemiological link between coffee intake and lower incidence of certain cancers [18]. CAF’s ability to inhibit or compete with xanthine oxidase (a ROS-generating enzyme of purine metabolism) has also been noted as a mechanism that could lower oxidative stress. Notably, CAF competes with the enzyme’s natural substrates (xanthine, hypoxanthine), which might reduce the production of pro-oxidant uric acid metabolites. However, these effects can vary: some studies report that coffee consumption modestly decreases serum uric acid (an anti-oxidant in plasma but pro-oxidant inside cells), while others find no effect—differences potentially attributable to variations in CAF source or genetic factors in metabolism. Overall, at modest doses, CAF anti-oxidant actions (direct and indirect) may help guard against the initiation of malignancies by mitigating oxidative DNA damage and mutagenesis.

In contrast, the pro-oxidant effects of CAF emerge more prominently at higher concentrations or in certain cancer cell contexts. High-dose CAF (in the millimolar range used in some in vitro studies) can increase intracellular ROS and interfere with DNA repair processes, thereby promoting oxidative damage within cancer cells [8,19]. Mechanistically, CAF at such doses inhibits ATM/ATR kinases—key regulators of the DNA damage response—which can force cancer cells through the cell cycle without repairing oxidative lesions, leading to apoptosis. This property has been exploited experimentally to enhance cancer cell kill in combination with DNA-damaging treatments. For example, in hepatocellular carcinoma cell lines, CAF (around 0.5–1 mM) significantly augmented the cytotoxicity of the chemotherapy drug 5-fluorouracil (5-FU). The combination led to synergistic inhibition of tumor cell proliferation and higher apoptosis rates compared to either agent alone. Notably, cancer cells treated with CAF plus 5-FU showed elevated ROS levels, along with upregulation of cleaved PARP and downregulation of anti-apoptotic Bcl-xL, indicating that CAF exacerbated oxidative stress to drive cell death. Similar pro-oxidant, anti-tumor effects of CAF have been observed in other models: CAF can deplete glutathione and increase ROS in breast cancer cells, sensitizing them to apoptosis [20]. In animal studies, CAF administration has been shown to inhibit tumor growth partly via disruption of tumor redox homeostasis; for instance, a recent study found CAF targets glucose-6-phosphate dehydrogenase (G6PD) in renal cell carcinoma cells, impairing the pentose phosphate pathway and tipping the redox balance toward oxidative stress to inhibit tumor growth [21]. These findings illustrate how CAF can function as a pro-oxidant within tumor cells, especially when used at higher doses or in combination therapy, thereby exerting anticancer activity.

Importantly, CAF anti-oxidant vs. pro-oxidant impact may differ between normal and cancerous cells. In normal cells or tissues, CAF ROS-scavenging and damage-mitigating effects can be protective—for example, low-dose CAF protected skin cells from UV- or chemical-induced oxidative senescence by activating autophagy via A2A receptor/SIRT3/AMPK signaling [22]. Paradoxically, the same anti-oxidant effect might aid tumor cell survival under stress: one report cautioned that while CAF protected normal skin from oxidative injury, it could also shield existing melanoma cells from ROS-induced damage, potentially supporting tumor growth under oxidative stress conditions. Thus, the context is critical—CAF might prevent cancer initiation by reducing oxidative DNA damage in healthy cells, yet in an established tumor, an anti-oxidant action could theoretically help cancer cells resist therapies that induce oxidative stress. Conversely, when CAF acts as a pro-oxidant at sufficient concentrations, it can selectively push cancer cells beyond their oxidative threshold, while normal cells (with better reserve anti-oxidant capacity) are less affected. In summary, CAF exhibits a dose-dependent redox duality: at lower doses or in combination with other coffee phytochemicals, it leans toward anti-oxidant, chemopreventive effects, whereas at higher doses or in therapeutic combinations, it can drive pro-oxidant, cytotoxic effects in cancer cells. Understanding this balance is key to leveraging CAF benefits while avoiding unintended protection of tumors.

### 4.1. Neuroendocrine Effects of Caffeine and Immune System Modulation in the Anticancer Therapy

CAF’s primary pharmacological action is the antagonism of adenosine receptors in the central nervous system and peripheral tissues. By blocking A1 and A2A adenosine receptors, CAF prevents adenosine (an endogenous neuromodulator) from inducing sedative and vasodilatory effects. The result is increased neuronal firing and neurotransmitter release, leading to heightened alertness and sympathetic nervous system activation. This neurostimulatory effect has significant endocrine consequences: CAF acutely triggers the release of catecholamine stress hormones. Human studies show that CAF ingestion elevates plasma epinephrine and norepinephrine levels, even at rest [3]. It also stimulates the hypothalamic–pituitary–adrenal (HPA) axis, resulting in increased secretion of cortisol. In one controlled trial, multiple doses of CAF (3 × 250 mg per day) raised cortisol concentrations in both men and women; CAF given before a stress challenge amplified the cortisol response compared to placebo. Thus, through central adenosine blockade, CAF mobilizes the body’s “fight-or-flight” hormonal response.

These neuroendocrine changes have direct immunological repercussions. Epinephrine and cortisol are well-known modulators of immune function, generally shifting immune responses toward a more anti-inflammatory or immunosuppressive profile during acute stress. Epinephrine, acting via β_2_-adrenergic receptors on immune cells, can transiently redistribute leukocytes (for example, increasing circulating natural killer cells), but it also alters cytokine production. In vitro and in vivo studies demonstrate that adrenergic stimulation suppresses the production of pro-inflammatory cytokines like tumor necrosis factor-α (TNF-α) and interleukin-12 while upregulating anti-inflammatory cytokines like IL-10 [5,23]. In a human endotoxemia experiment, a short infusion of epinephrine dramatically inhibited LPS-induced TNF-α release and concurrently increased IL-10 levels in blood, indicating a net anti-inflammatory effect of acute adrenaline exposure. Cortisol, a glucocorticoid, likewise broadly dampens immune activity—it inhibits T-cell proliferation and cytokine secretion and is often therapeutically used to counteract overactive immune responses. CAF-induced elevation of cortisol, therefore, may impose a mild immunosuppressive influence. Indeed, some animal studies suggest that high doses of CAF can reduce lymphocyte proliferation and alter cytokine balances, consistent with cortisol’s effects [24]. Taken together, the acute hormonal surge from CAF tends to restrain excessive inflammatory responses—an effect that might be beneficial in preventing cytokine-mediated damage but could be detrimental if a strong immune attack on tumor cells is needed.

On the other hand, CAF’s direct actions on immune cells can counteract immunosuppression in the tumor microenvironment. By blocking A2A adenosine receptors, CAF can prevent adenosine-mediated immune inhibition. This is particularly relevant in cancer, where tumors often create an adenosine-rich milieu that suppresses T-cell and NK cell activity via A2A receptor signaling (elevating cAMP and driving T-cells toward a “exhausted” or less cytotoxic state) [25]. CAF adenosine antagonism lifts this brake on immune cells. Preclinical studies have shown that CAF can promote a more pro-inflammatory, anti-tumor immune profile. In a mouse model of carcinogen-induced fibrosarcoma, continuous administration of CAF (0.1% in drinking water) significantly protected against tumor development: only 14% of CAF-treated mice developed tumors versus 53% of control mice [26]. The CAF-consuming mice that remained tumor-free exhibited signs of heightened immune activity, such as leukocyte infiltration at tumor challenge sites and even autoimmunity (alopecia), indicating a potent immune response. Immune assays confirmed that CAF-treated mice mounted stronger Th1-type responses; their lymphocytes released higher levels of pro-inflammatory cytokines (e.g., interferon-γ) in reaction to tumor antigens compared to non-treated mice. Remarkably, knocking out the A2A adenosine receptor in mice produced a similar enhancement of anti-tumor immunity, and in both cases (CAF treatment or A2A gene deletion), the outcome was tumor growth suppression or rejection. These results support the idea that CAF immunological benefit in cancer is largely through antagonizing A2A receptors on immune cells, thereby preventing adenosine from “putting the immune system to sleep” in the tumor setting.

Given these opposing facets—stress-hormone-mediated dampening of immunity versus direct relief of adenosine-based immunosuppression—the net effect of CAF on the immune system in cancer can be complex. Timing and context likely determine whether CAF synergizes with or antagonizes anti-tumor immunity. Synergy with immunotherapy: When an immune response is being actively harnessed to fight cancer (as with immunotherapies), CAF blockade of adenosine A2A receptors may enhance T-cell activity. Pioneering work by Ohta et al. showed that pharmacological A2A antagonists, such as CAF, improved T-cell-mediated tumor destruction in mice. In that study, adding CAF or other A2A blockers allowed cytotoxic T lymphocytes to overcome the suppressive tumor microenvironment, leading to greater tumor inhibition and even eradication of metastases. Similarly, in adoptive T-cell transfer models, co-treatment with CAF increased the survival of mice compared to T-cell therapy alone, implying a cooperative effect between CAF and immune-based cancer therapy. These findings have driven interest in adenosine signaling as a target in cancer therapy—several clinical trials are now investigating selective A2A receptor antagonists to combine with checkpoint inhibitors or cellular therapies [27]. CAF, as a non-selective adenosine antagonist, essentially functions as a crude prototype of these drugs. Its ubiquitous consumption has even been considered a potential confounder in trials: patients who drink a lot of coffee might inadvertently receive an immune-boosting adenosine blockade, possibly skewing the outcomes of immunotherapy trials or masking the benefits of experimental A2A inhibitors [28]. On balance, the adenosine-blocking action of CAF is pro-immune and would be expected to synergize with immunotherapies by unleashing T-cells and NK cells that would otherwise be suppressed by tumor-derived adenosine.

One concern is that CAF systemic effects (like elevated cortisol and adrenaline) might transiently inhibit certain immune functions that immunotherapies aim to stimulate. For example, checkpoint inhibitor treatments rely on reinvigorating T-cells; if a patient is highly caffeinated and experiences repeated cortisol spikes, in theory, this might blunt T-cell activation or proliferation to a small degree. So far, there is little direct clinical evidence that normal dietary CAF intake impairs cancer immunotherapy—any such effect is likely subtle. Some in vitro studies note immunosuppressive effects of CAF on lymphocytes (e.g., high CAF can reduce T-cell IL-2 secretion and proliferation) [24], which could be detrimental if it occurred in vivo. However, the concentrations at which CAF directly suppresses lymphocyte function are typically high. In physiological conditions, the pro-immune adenosine blockade seems to dominate over any mild HPA axis immunosuppression. Indeed, observational data in humans generally associate coffee intake with better cancer outcomes, not worse. It is possible that excessive CAF in an already stressed patient could add to immunosuppressive stress hormone levels, but moderate consumption might have net neutral or positive effects. In summary, CAF appears more likely to act synergistically with cancer immunotherapy by counteracting adenosine-mediated immunosuppression and promoting a Th1-biased response. Any antagonistic effects via stress hormones are probably transient and outweighed by CAF immune stimulation, though this balance may vary individually.

### 4.2. Caffeine and Its Influence on the Appetite

There are numerous studies regarding the influence of CAF on appetite. However, the available research provides mixed results; thus, it is hard to clearly assess its properties [29]. One of the studies on mice suggests that CAF increases food intake and reduces anxiety-related behaviors. The authors measured 2h and 24h food intake and body weight during daily administration of CAF and examined the results. They have found out that administration led to a higher food intake in a dose-dependent manner, with lower doses of CAF having a greater effect on enhancing appetite compared to higher doses [30].

On the other hand, Gavrieli A. et al. conducted a crossover study in which healthy men received either caffeinated or decaffeinated coffee with breakfast. Energy intake and appetite did not differ significantly between the groups, suggesting that CAF had no notable effect in this context. However, a significant increase in serum cortisol levels was observed following the caffeinated coffee intervention [31].

Other studies discovered that neither total intake nor appetite was significantly different by CAF treatment. Furthermore, there were no significant interactions between CAF treatment and intake in or out of the laboratory [32].

Evidence regarding the influence of CAF on appetite, appetite sensations, and energy intake remains equivocal. Therefore, it is crucial to carry out further research and widen the knowledge in the area in order to clinically address the patients’ needs. What is more, there are no studies on oncology patients, especially cachectic ones. Such studies could improve the treatment and well-being of those patients.

### 4.3. Caffeine and Its Influence on Cancer Pain

Pain in cancer is a major clinical challenge that significantly affects patients’ quality of life. Although opioids are crucial in cancer pain management, they are often insufficient to fully control severe or refractory pain. Therefore, exploring strategies to enhance their effectiveness is of great therapeutic interest. One study assessed the efficacy of CAF infusion as an adjuvant analgesic to opioid therapy in patients with advanced cancer. The CAF group experienced a mean reduction in pain intensity of 0.833 (95% confidence interval [CI] 0.601–1.066), compared to 0.350 (95% CI 0.168–0.532) in the placebo group. What is more, drowsiness showed a significant improvement in the CAF group after the first trial (*p* = 0.041). However, the results did not reach clinical significance [33]. Further studies are essential to evaluate the influence of CAF on cancer pain and opioid therapy, as it seems to have therapeutic potential in this area.

### 4.4. Cancer-Related Fatigue

Cancer-related fatigue (CRF) affects a large number of cancer survivors both during and after their treatment. CRF has a substantial negative impact on day-to-day functioning and the quality of life. Nevertheless, this issue is often overlooked by health care professionals, and there is currently no gold standard when it comes to treatment.

Unfortunately, it is currently not well understood if CAF plays a role in the CRF treatment. A 2015 randomized, placebo-controlled, double blind study conducted on prostate cancer survivors proved that the administration of 6 mg of anhydrous CAF per kg 1 h before the training session resulted in enhanced exercise capacity and muscle strength. However, post-exercise fatigue and its perception were comparable between the test and placebo groups [34]. A more recent study examined the effects of CAF on tumor-bearing mice. BALB/c mice were injected with C26 colon carcinoma cells and divided into two groups: the first group was fed with a normal diet, and the diet of the second group was enriched with 0.05% CAF. Mice on a CAF-enriched diet achieved improved running performance that was measured on a treadmill. Researchers suppose that this effect was caused by lipolysis promotion, restoring non-esterified fatty acids, which can serve as an alternative energy source [35]. However, higher-quality studies are needed to determine whether the CAF is suitable for the treatment of cancer-related fatigue.

### 4.5. Caffeine Interactions with Chemotherapeutic Agents

It is indicated that CAF may increase the efficacy of chemotherapeutic agents by influencing the cell cycle, triggering apoptosis, and reducing drug efflux from cancer cells [20]. Earlier studies suggested fine reduces the cytotoxic potential of doxorubicin by lowering the free drug concentration.

Recent evidence supports the potential of CAF to enhance the efficacy of chemotherapeutic agents through multiple mechanisms, including inhibition of cell cycle checkpoints, promotion of apoptosis, and reduction in drug efflux. As highlighted in [36], CAF acts as a chemosensitizer by modulating DNA damage response pathways and enhancing the susceptibility of cancer cells to agents such as cisplatin and doxorubicin. The study emphasizes the relevance of CAF interference with ATR-Chk1 signaling, thereby abrogating G2/M arrest and promoting mitotic catastrophe in tumor cells. These findings strengthen the rationale for exploring CAF as an adjuvant in combination cancer therapies.

In 2023, we examined the effects of CAF combined with doxorubicin and oxaliplatin on B16F10 cells. Results demonstrated that CAF increased the cytotoxicity of both [37].

In another study, CAF was combined with docetaxel and administered to MCF-7 breast cancer cells. Once more, CAF potentiated the cytotoxic effect of chemotherapeutics and induced the cancer cell autophagy and apoptotic protein levels [38].

CAF is known to interfere with DNA damage-induced checkpoint activation, particularly at the G2/M transition, making it a potential chemosensitizer. As shown by Bode et al., CAF abrogates G2/M arrest and enhances apoptosis when used in combination with DNA-damaging agents such as cisplatin or camptothecin. This synergistic effect is attributed to CAF inhibition of ATM/ATR kinases, leading to unscheduled mitotic entry and subsequent cell death [8]. Such findings highlight CAF’s promise in potentiating the efficacy of conventional chemotherapeutics.

In efforts to overcome multidrug resistance (MDR), CAF has also been investigated as a component of nanoparticle-based drug delivery systems. A study by Merlin et al. demonstrated that poly(lactic-co-glycolic acid) (PLGA) nanoparticles co-loaded with ursolic acid and CAF enhanced drug delivery and cytotoxicity in resistant cancer cell lines. CAF acted not only as a bioactive compound but also contributed to the inhibition of drug efflux mechanisms, thereby improving intracellular drug accumulation [39]. These findings suggest that CAF may serve as a promising adjuvant in nanocarrier-based strategies to sensitize resistant tumors to chemotherapy. These findings highlight CAF’s potential to augment current chemotherapeutic treatments and support the development of novel cancer therapies.

### 4.6. Temperature of Caffeinated Beverages and Esophageal Risk

Beyond the biochemical effects of CAF, the temperature at which coffee and other beverages are consumed has emerged as a significant factor influencing the risk of esophageal cancer. Epidemiological studies have consistently demonstrated that regular consumption of very hot beverages—including coffee, tea, and maté—is associated with an increased risk of esophageal squamous cell carcinoma (ESCC) [40].

Mechanistically, the ingestion of hot liquids can cause thermal injury to the esophageal mucosa, leading to chronic inflammation and repeated cycles of tissue damage and repair. These processes may promote DNA damage and abnormal cell proliferation, which are key events in carcinogenesis [41].

A comprehensive meta-analysis encompassing 39 observational studies with over 42,000 participants found that individuals who regularly consumed hot beverages and foods had a significantly increased risk of ESCC (odds ratio [OR] = 1.60; 95% confidence interval [CI]: 1.29–2.00), while no significant association was observed for esophageal adenocarcinoma (EAC) (OR = 0.79; 95% CI: 0.53–1.16) [42].

Further supporting this association, a case–control study conducted in southern China reported that individuals consuming hot beverages exhibited a markedly increased risk of esophageal cancer. Specifically, those who drank hot beverages had an OR of 4.13 (95% CI: 2.13–8.05), and those consuming very hot beverages had an OR of 8.55 (95% CI: 3.67–20.9), compared to individuals who consumed beverages at lower temperatures [43].

The International Agency for Research on Cancer (IARC) has also emphasized the carcinogenic potential of consuming beverages at high temperatures. In their evaluation, they classified the consumption of very hot beverages (above 65 °C) as “probably carcinogenic to humans,” citing evidence that high-temperature liquids can cause thermal injury to the esophageal mucosa, leading to increased cancer risk [44].

Collectively, these studies reinforce the importance of moderating the temperature of consumed beverages as a practical measure to reduce the risk of ESCC. Allowing hot drinks to cool to below 60 °C before consumption is a simple yet effective strategy for mitigating this risk. 

Table 1 summarizes key studies investigating the role of caffeine (CAF) in cancer research.

## 5. Influence on Psychiatric Disorders

### 5.1. Depression

As a non-selective antagonist of adenosine receptors, CAF exhibits anti-inflammatory effects and may reduce the risk of psychiatric and neurological disorders. In a study conducted on Sprague Dawley rats, CAF improved neuroinflammatory responses and depressive behaviors induced by lipopolysaccharides (LPS). Its mechanisms included the regulation of phosphorylation of protein kinase B (AKT) and nuclear factor κB (NF-κB). These findings suggest CAF could be a potential preventative agent against inflammatory conditions [45]. Furthermore, another study found that CAF use disorder (CUD) and withdrawal symptoms are positively correlated with levels of depression, anxiety, and stress (DASS) in adults. Participants, with an average age of 27.8 (SD = 7.8) years, consumed 461.21 (SD = 11.09) mg of CAF daily. The development of CUD and CAF withdrawal exacerbated DASS levels, highlighting the need for public health interventions [46]. Another research study discussed CUD in patients. CUD was identified in 19.5% of participants (more common in men at 25.08%) with an average daily CAF intake of 146.67 mg, and CAF withdrawal symptoms were present in 46.62% of respondents. In addition, commonly reported harms included sugar cravings (42.9%) and insomnia (39.3%). These results emphasize the need for evidence-based therapeutic strategies to manage those disorders [47].

Habitual CAF consumption increases cortisol response to psychosocial stress, whereas acute consumption does not have this effect. The “masking” of depression risk factors was observed in individuals who abstain from CAF. This suggests that habitual CAF intake may play a protective role against depression by influencing cortisol secretion [48]. According to the analysis of the National Health and Nutrition Examination Survey (2007–2016), moderate CAF consumption (119.5–236.5 mg per day) can reduce depressive symptoms in individuals without cancer. In contrast, no such relationship was observed among cancer patients [49]. Moreover, another study involving students from Taibah University showed that despite high levels of stress, anxiety, and depression, there was no significant association between CAF consumption and these symptoms. These findings suggest that CAF consumption does not harm students’ mental health [50]. Furthermore, early morning CAF intake is associated with a lower prevalence of depression compared to abstaining from CAF in the morning. This suggests that the timing of CAF intake may play an essential role in adult mental health [51]. Notably, a relationship between coffee consumption and postpartum depression symptoms was found. According to one of the recent studies, consuming more than three cups of coffee daily could reduce the risk of postpartum depression, especially in women who were not breastfeeding during the first 1–2 years after delivery. However, the link between decaffeinated coffee consumption and depression remains unclear [52]. Therefore, more detailed research is still needed to assess the knowledge in this area and potentially use CAF as an adjunct to psychotherapy and psychiatric treatment.

### 5.2. Anxiety and Aggression Disorders

A 2024 meta-analysis showed that CAF consumption increases the risk of anxiety, especially at doses exceeding 400 mg per day. Lower doses of CAF moderately increase anxiety risk, as higher doses significantly elevate it. These findings suggest that CAF may be associated with a heightened risk of anxiety in healthy individuals [53]. A study conducted on 46,873 adolescents in South Korea found that high consumption of CAF-containing beverages is associated with higher levels of anxiety. This relationship was evident among high school students and individuals with shorter sleep durations. The effect intensified with increased anxiety levels, demonstrating a dose-dependent relationship. The association between CAF consumption and anxiety remained statistically significant regardless of gender or socioeconomic factors [54].

Additional details on CAF effects on the nervous system, cognitive function, long-term health outcomes, and therapeutic limitations are presented in Table 2.

## 6. Effects on the Nervous System

### 6.1. Parkinson’s Disease

CAF interacts with specific genetic variants such as MAPT, SLC2A13, LRRK2, ApoE, NOS2A, GRIN2A, CYP1A2, and ADORA2A, which may influence the risk of developing Parkinson’s Disease (PD). Preliminary studies suggest positive effects of these interactions; however, further analyses are needed to confirm these findings and better understand the role of genes and CAF in the development of PD [55]. Recent research indicates that drinking coffee, primarily due to its CAF content, may reduce the risk of developing PD because of its neuroprotective properties. Furthermore, other coffee components, such as chlorogenic acid and cafestol, also exhibit anti-Parkinsonian effects. Additionally, coffee’s impact on gut microbiota may protect against inflammation and misfolded protein aggregation [56]. One of the studies demonstrated that CAF supplementation significantly improved motor functions in patients with PD (80% compared to 16.7% in the placebo group) with minimal side effects. Patients receiving CAF showed improvement in motor assessments (UPDRS III), suggesting the potential benefits of CAF as an adjunct therapy for PD patients [57]. Moreover, there is more research regarding regular coffee consumption, specifically CAF and its metabolites (paraxanthine and theophylline), and PD risk. In a prospective study, patients with the highest coffee consumption had a 37% lower risk of PD. Case–control studies also associated previous CAF and metabolite levels with a reduced risk of PD. These findings indicate that CAF has neuroprotective effects in the context of PD [58].

Notably, the effects of CAF and haloperidol (a selective antagonist of the D2 receptor) on fatigue in a reserpine-induced mouse model of PD were assessed. The results showed impaired motor control and fatigue in mice, alleviated by L-DOPA and a combination of CAF and haloperidol. The findings suggest that A2AR-D2R antagonism could have therapeutic potential in managing fatigue in PD patients [59].

Additionally, adenosine plays homeostatic and neuromodulatory roles in the brain, primarily on A1 and A2A receptors. A2A receptor antagonists (A2AR), such as istradefylline, are promising therapies for patients with PD and traumatic brain injuries (TBIs), particularly regarding neuroprotection and cognitive function improvement. Studies indicate that the A2AR receptor plays a role in neurotoxicity and cognitive deficits in PD and TBI. Epidemiological evidence also suggests that CAF consumption reduces the risk of PD and cognitive decline [60].

### 6.2. Migraine

CAF consumption, whether daily or occasional, has a significant biological impact on the nervous system. CAF affects headaches, particularly migraines, although the mechanisms of this action are not yet fully understood. Despite the introduction of new migraine medications, such as gepants and ditans, a combination of paracetamol and CAF (1000 mg/130 mg) remains a valuable option for treating acute migraine attacks, particularly for patients over 65 years old, individuals with cardiovascular conditions, those unresponsive to new drugs, as well as pregnant, and breastfeeding women. CAF enhances the analgesic effect of paracetamol, making this combination effective for migraine treatment [61]. The FDA has approved the combination of acetylsalicylic acid (250 mg), paracetamol (200 mg), and CAF (50 mg) as safe and effective for treating acute headaches, including migraines. It is well-tolerated for tension-type headaches and taken orally to treat acute migraine attacks with minimal side effects [62]. A large cross-sectional study found that while coffee consumption was slightly more common among individuals with migraine than those with non-migraine headache or no headache, it was not significantly associated with headache characteristics or response to acute treatment. Despite the observed links with stress and psychiatric comorbidities, coffee intake did not meaningfully influence migraine severity or treatment outcomes [63].

Furthermore, excessive consumption may lead to medication-overuse headaches. Notably, abrupt CAF withdrawal can also trigger migraines [64]. Recent evidence highlights CAF’s dual role in headache disorders—offering acute relief in some cases while potentially worsening or triggering headaches with high intake, abrupt withdrawal, or inconsistent use—supporting a balanced, individualized approach to CAF consumption [65]. In 17 studies, 2% to 30% of participants identified CAF or its withdrawal as a migraine trigger. Therefore, moderate CAF consumption and limited use of CAF-containing medications are recommended for mild headaches. However, there is insufficient evidence to recommend complete CAF withdrawal. Excessive CAF intake may lead to chronic migraines, and limiting consumption to 200 mg per day is advised [66]. For other types of headaches, such as cluster headaches, post-lumbar puncture headaches, or idiopathic intracranial hypertension, CAF serves as an essential therapeutic agent. A study revealed that 77.5% of children and adolescents with migraines had detectable CAF levels in their blood and urine. Patients with higher CAF levels scored significantly higher on the HIT-6 test, indicating greater headache severity. CAF consumption exacerbated migraine symptoms in children and adolescents [67]. A study published in 2019 found that the consumption of caffeinated beverages may influence the occurrence of migraines. Among 98 individuals with episodic migraine, a nonlinear relationship was observed between the amount of CAF consumed and the risk of headache—higher intake was associated with an increased risk of migraine. This effect also depended on individual habits and the use of hormonal contraceptives. The results suggest that high doses of CAF may trigger migraines, while moderate amounts may not have a negative impact [68].

### 6.3. Stroke

The relationship between CAF consumption and stroke risk is complex, with studies presenting both protective and adverse associations. While some research suggests that regular coffee intake may reduce stroke risk, other studies indicate a transient increase in risk following acute consumption, particularly among infrequent drinkers.

A multicenter case-crossover study by Mostofsky et al. found that the risk of ischemic stroke was transiently elevated in the hour following coffee consumption, especially among individuals who typically consumed one or fewer cups per day. The relative risk (RR) of stroke in the hour after consuming coffee was 2.0 (95% confidence interval [CI]: 1.4–2.8; *p* < 0.001). Importantly, this increased risk was not observed in habitual coffee drinkers, and no significant gender differences were noted in the study [69].

In contrast, the Health Examinees (HEXA) study in Korea, involving approximately 146,830 participants aged 40 to 69, indicated an inverse relationship between coffee consumption and stroke risk among women [70]. Higher coffee intake (≥3 cups/day) was associated with a 38% lower odds ratio for stroke in women (OR: 0.62; 95% CI: 0.47–0.81; *p* for trend < 0.0001). No significant association was found among men in the study.

A meta-analysis encompassing over 2.4 million participants found that individuals consuming three to four cups of coffee daily had a 21% lower risk of stroke compared to those with minimal or no coffee intake [71].

Further evidence comes from a cross-sectional study analyzing data from the National Health and Nutrition Examination Survey (NHANES) between 2009 and 2014 [72]. This study found that higher urinary levels of CAF and its metabolite paraxanthine were associated with a lower risk of stroke, especially among Mexican American participants. Specifically, higher urinary CAF levels were associated with a lower risk of stroke in Mexican Americans (odds ratio [OR] = 0.886, 95% CI: 0.791–0.993, *p* = 0.037), and higher urinary paraxanthine levels were associated with a lower risk of stroke incidence (OR = 0.991, 95% CI: 0.984–0.999, *p* = 0.027).

In summary, while habitual coffee consumption may offer protective benefits against stroke, particularly in certain populations, acute intake among infrequent drinkers could transiently increase the risk. These findings underscore the complexity of the relationship between CAF and cerebrovascular health, highlighting the need for individualized recommendations based on consumption patterns and demographic factors.

## 7. Effects on Cognitive Functions

### 7.1. Influence on Dementia

Numerous studies suggest that CAF consumption reduces the prevalence of dementia. Increased CAF intake among white women aged 65–80 years decreased the likelihood of dementia or cognitive impairment. Drinking moderate amounts of coffee (3–5 cups per day) also reduced dementia prevalence compared to non-coffee drinkers. Among coffee drinkers, dementia was less common in women than in men. Additionally, a study conducted later in life found that individuals who rarely drank coffee were more likely to experience depression (based on the Beck Depression Inventory) compared to moderate coffee consumers [73]. A study involving residents from a retirement community in California revealed that individuals who consumed over 100 mg of CAF daily at the age of 61–80 significantly reduced their risk of dementia. Furthermore, retrospective observational studies suggest lifelong coffee consumption improves cognitive functions in older women, although this relationship was not observed in older men [74]. Regular CAF consumption may reduce the risk of developing Alzheimer’s disease. Although no conclusive evidence exists, decades of research suggest that coffee and CAF may offer neuroprotective benefits by enhancing short-term cognition and potentially reducing long-term cognitive decline. While in vitro and animal studies support this link, epidemiological findings remain mixed, and further clinical trials are needed to confirm these potential preventive effects [75]. What is more, a study conducted by the CAIDE found that drinking 3–5 cups of coffee per day in midlife was associated with a 65% reduced risk of dementia and Alzheimer’s disease in later life [76]. While findings across studies are somewhat inconsistent, the majority support coffee’s potential protective effects, possibly due to CAF, antioxidants, or improved insulin sensitivity. This study provides molecular evidence that espresso coffee and its components, such as CAF and genistein, may exert neuroprotective effects by inhibiting tau protein aggregation—a key mechanism in the development of Alzheimer’s disease [77]. One study examined the relationship between CAF consumption and cognitive impairment in two cohorts. In the Rush Memory and Aging Project, higher CAF intake (>100 mg/day) was associated with a greater risk of dementia and Alzheimer’s disease. In contrast, in the UK Biobank, moderate consumption (100–400 mg/day) was linked to a lower risk of dementia. CAF also demonstrated an inverse relationship with the presence of Lewy bodies in postmortem brain tissues [78].

### 7.2. Influence on Alzheimer’s Disease

Alzheimer’s disease (AD) is the leading cause of dementia, with projections estimating 131.5 million cases by 2050 [79]. While clinical and preclinical studies largely suggest CAF may have neuroprotective effects in AD, especially in animal models, more rigorous human trials are needed to confirm these findings. A large prospective cohort study involving over 365,000 UK adults found that moderate consumption of coffee and tea—either separately or in combination—was associated with a reduced risk of stroke, dementia, and poststroke dementia [80]. The lowest risk was observed with 2–3 cups of coffee and 2–3 cups of tea per day, resulting in up to a 32% lower risk of stroke and 28% lower risk of dementia. A nonlinear dose–response relationship was noted, and the protective effect was particularly evident for ischemic stroke and vascular dementia. In another study, a linear relationship between coffee consumption and dementia risk was observed only in slower CAF metabolizers [81]. Drinking ≥4 cups per day decreased dementia risk in individuals with the C allele, whereas faster metabolizers experienced increased risk with consumption exceeding 3 cups per day.

What is more, another study revealed that lower CAF intake is associated with a higher risk of memory disorders and lower levels of amyloid biomarkers in cerebrospinal fluid in patients with mild cognitive impairment (MCI) and AD. CAF consumption may influence these patients’ memory and biomarkers [82]. Furthermore, a study found that regular consumption of matcha improved emotion perception and showed a trend toward better sleep quality in older adults with mild cognitive impairment. However, no significant improvements were observed in core cognitive function measures [83]. A meta-analysis indicated that consuming 2.5 cups of coffee daily reduces the risk of Alzheimer’s disease, while one cup of tea daily reduces the risk of cognitive impairment by 11%. Appropriate amounts of coffee and tea can effectively lower the risk of dementia [84].

### 7.3. Cognitive Functions

One study found that while CAF increases brain activity related to working memory (WM) in healthy adults, it does not enhance behavioral performance. In a double-blind, randomized, crossover trial, 20 young adults consumed CAF or a placebo for 10 days [85]. Working memory tasks and fMRI scans revealed that daily CAF intake increased cerebral metabolic demand and reduced hippocampal activity, which may impair performance and reflect CAF-induced plasticity. These findings highlight the need for more nuanced assessments of CAF impact across different populations.

Furthermore, acute CAF intake at a dose of 6 mg/kg improves reaction time in healthy, physically active young adults but also increases perceived activeness and nervousness. No significant effects were observed on anticipation, sustained attention, or memory [63]. A study examining the effects of creatine nitrate and CAF on resistance-trained athletes found that combined supplementation significantly improved cognitive performance (Stroop test) without enhancing exercise outcomes. No adverse effects were reported during the seven-day intervention. The combination was more effective for cognitive enhancement than CAF alone [86]. Another study investigated the effects of combined CAF and fish collagen peptides (C&F) on cognitive function in sleep-deprived mice. C&F improved cognition in a dose- and time-dependent manner, protected hippocampal tissue from sleep deprivation-induced damage, and reduced pro-inflammatory neurometabolite levels. Microbiota analysis revealed increased Lactobacillus murinus and modulation of metabolic pathways, suggesting that C&F alleviates cognitive impairments via the gut–microbiota–brain axis [87].

A study found that a single 400 mg dose of CAF improved functional capacity (up-and-go and sit-to-stand tests) and cognitive performance (reaction time and vigilance) in patients with end-stage renal disease (ESRD). No significant changes were observed in postural balance [88]. This study, based on NHANES 2011–2014 data, investigated the role of alanine aminotransferase (ALT) in the relationship between CAF metabolites and cognitive function in older adults using verbal fluency testing. Bayesian regression identified 3,7-dimethylxanthine as positively associated with cognitive performance, while low ALT levels were linked to cognitive decline. However, ALT did not mediate the association between CAF metabolites and cognitive function. A study on elite e-sports players showed that CAF supplementation at 3 mg/kg significantly enhanced reaction times, cognitive performance (Stroop task, visual search), and shooting abilities (kill ratio, hit accuracy, and time to target) compared to placebo. These findings suggest that moderate CAF intake can improve both cognitive and motor aspects of performance in competitive gaming [89].

Another study found no association between CAF consumption and survival or progression of ALS. However, patients with the C/T and T/T rs2472297 genotype showed better cognitive performance with higher CAF intake. This suggests that CAF may positively affect cognitive functions in fast CAF metabolizers without impacting disease progression [90].

A study evaluating the effects of a low-CAF beverage (“WKUP GT”) containing extracts from carob, guarana, green tea, and elderberry found improved attention, memory, and executive functions after lunch compared to a placebo. Significant improvements were observed in visual information processing and multitasking tests without affecting blood pressure or heart rate, making this drink an effective alternative to CAF [91].

## 8. Long-Term Caffeine Consumption and Its Effects

It is widely believed that CAF is an addictive substance. Researchers examine CAF as a potentially addictive substance, highlighting characteristics such as withdrawal symptoms, a craving to limit use, and continued consumption despite harm. While CAF does not meet all the classical criteria for addiction, it is considered a substance with abuse potential due to its widespread use and individual variability in sensitivity [92]. Heinz et al. argue that withdrawal symptoms and increased tolerance are insufficient criteria for classifying CAF as an addictive substance. These symptoms are likened to the sudden discontinuation of effective medications, such as beta-blockers or anticoagulants, which are not considered addictive but can disrupt homeostasis. Similarly, withdrawal symptoms from antidepressants like selective serotonin reuptake inhibitors (SSRIs) are presented as another example. CAF acts as a competitive agonist of adenosine receptors without inducing dopamine release in the ventral striatum [93]. Studies indicate that chronic CAF consumption does not impair the ergogenic response to acute CAF intake, although some findings suggest reduced effectiveness in regular CAF consumers [94].

A review by Tallis et al. indicates that CAF may have an ergogenic effect regardless of typical consumption habits. Concerns about the toxicity of high CAF doses have led to media reports of deaths related to CAF overdose. The article detailed a fatality resulting from “miscalculated, massive overdosing” involving powdered CAF [95]. The literature describes five CAF-related deaths (5%) among athletes, including two amateur bodybuilders, a basketball player, and a wrestler, with ages ranging from 18 to 44 years. Cardiac arrest caused by ventricular fibrillation was the cause of death in all cases.

A review by Willson et al. identified ventricular fibrillation as the most common cause of death, with coronary artery spasms also suggested as a cause in myocardial infarction cases [96].

Reduced benefits observed in some studies may be related to CAF doses administered during performance trials, corresponding to typical consumption levels (~3 mg/kg/day). Pickering et al. suggest that habitual CAF consumers may require 6 mg/kg or more doses to achieve ergogenic effects [97]. Zhang et al. found that lower CAF doses (≤3 mg/kg) suffice for central nervous system (CNS) effects, whereas higher doses (6–9 mg/kg) may be needed for peripheral effects [98].

## 9. Limitations and Risks of Therapeutic Caffeine Use

Despite compelling preclinical evidence that CAF can modulate DNA repair pathways, oxidative stress, endothelial function, and key signaling cascades to sensitize tumor cells to chemotherapeutics, its translation into a viable anticancer adjuvant is beset by several critical barriers. The concentrations of CAF demonstrating cytotoxic or chemosensitizing effects in vitro (typically 100–500 µM) far exceed those achieved by customary dietary intake (<10 µM after a 200 mg coffee dose), and no consensus exists on how to safely escalate to “therapeutic” plasma levels in humans without provoking toxicity [1]. Although regulatory agencies recognize that moderate consumption (≤400 mg/day) is well tolerated in healthy adults, exceeding this threshold—particularly via concentrated pharmaceutical formulations—can precipitate adverse events [1]. Inter-individual CAF clearance varies markedly owing to CYP1A2 genetic polymorphisms, hepatic function, concomitant medications (e.g., CYP1A2 inhibitors), and lifestyle factors such as smoking. These influences can extend CAF half-life from roughly 2–3 h up to 12 h, thereby increasing the risk of accumulation and consequent cardiovascular or central nervous system toxicity in vulnerable populations, including those with cardiovascular disease, pregnancy, or psychiatric comorbidities [1]. At supraphysiological doses—far above dietary levels—CAF stimulant properties may transition from therapeutic to deleterious. High plasma concentrations have been implicated in tachyarrhythmias, seizures, hypokalemia, lactic acidosis, and even fatal outcomes when pure CAF powders are misused; the median lethal dose in humans is estimated at 150–200 mg/kg, with documented fatalities at doses as low as 57 mg/kg—levels unattainable through beverages alone but conceivable with potent supplement formulations [99]. Even modest overdosing in these contexts can trigger restlessness, tremors, insomnia, and serious cardiac disturbances that require urgent medical intervention [99]. Finally, human trials exploring CAF’s impact on appetite, fatigue, and its interaction with standard chemotherapy regimens remain few and underpowered, often yielding contradictory outcomes. Rigorous, placebo-controlled, randomized studies are, therefore, essential to define a safe and efficacious dosing window, characterize pharmacodynamic biomarkers (e.g., DNA damage markers), stratify participants by metabolic genotype, and monitor for CAF intoxication under drug-level exposures, so that any therapeutic benefit can be responsibly assessed without unacceptable toxicity.

## 10. Conclusions

Long-term CAF consumption exerts a dualistic profile of effects that hinge critically on both dosage and individual susceptibility. At moderate levels—up to approximately 400 mg per day in healthy adults— CAF reliably enhances alertness, reaction time, and certain domains of executive function while also displaying neuroprotective properties that appear to mitigate the risk or slow the progression of neurodegenerative disorders. In oncology models, low concentrations of CAF have been shown to act as antioxidants that may contribute to cancer prevention, whereas higher, pharmacological doses can induce oxidative stress and drive apoptotic cell death in tumor cells. Moreover, CAF capacity to elevate cortisol modestly, block A2A adenosine receptors, and boost T-cell and natural killer cell activity has demonstrated tumor-protective effects in vivo, suggesting possible synergy with immunotherapeutic regimens.

Conversely, intake at higher doses can precipitate a spectrum of adverse outcomes: heightened anxiety, sleep fragmentation, and, in vulnerable individuals, cardiovascular disturbances, including transient hypertension and, in rare, extreme cases, ventricular arrhythmias or cardiac arrest due to overdose. These stimulant-related risks are amplified when CAF is delivered in concentrated forms—such as energy drinks or dietary supplements—where inadvertent overuse is common. Clinicians should, therefore, exercise caution when advising populations with heightened sensitivity—adolescents, pregnant women, those with anxiety disorders or preexisting cardiac conditions—as even moderate exposure may unmask serious side effects.

Given the pervasive and habitual nature of CAF use, further longitudinal and mechanistic studies are imperative. Well-powered clinical trials that integrate pharmacokinetic monitoring, genetic stratification, and comprehensive safety assessment will be essential to define safe, efficacious dosing windows for both neuroprotective and anticancer applications. Ultimately, such research will enable personalized guidance that fully leverages CAF’s therapeutic potential while minimizing its risks.

## Figures and Tables

**Table 1:** Summary of studies on the effects of caffeine in cancer research.

Area of Interest	Effect of Caffeine	Cited Studies
DNA Damage Response and Cell Cycle Regulation	Inhibits ATR-Chk1 signaling, sensitizing cancer cells to DNA-damaging agents by impairing DNA repair and promoting mitotic catastrophe.	[8,9,10,11]
Cancer Treatment	Low doses: anti-oxidant protection and cancer prevention. High doses: oxidative stress and enhanced cell death.	[8,18,19,20,21,22]
Neuroendocrine Effects and Immune System Modulation	CAF modulates immune response by elevating cortisol (mild immunosuppression) and blocking A2A adenosine receptors, enhancing T and NK cell activity. Demonstrated tumor protection in vivo; may synergize with immunotherapy.	[5,23,24,25,26,27,28]
Influence of Caffeine on the Appetite	Mixed findings; possible appetite increase in animals (dose-dependent), no significant effect in humans; CAF may raise cortisol levels.	[29,30,31,32]
Caffeine and Cancer Pain	Modest reduction in pain and drowsiness as an adjuvant to opioids; not clinically significant.	[33]
Cancer-Related Fatigue	Improved physical performance and muscle strength; no significant effect on perceived fatigue	[34,35]
Caffeine Interactions with Chemotherapeutic Agents	Enhances cytotoxicity of agents like doxorubicin, oxaliplatin, and docetaxel via apoptosis/autophagy. Additionally, abrogates G2/M checkpoint arrest by inhibiting ATM/ATR, sensitizing cancer cells to DNA-damaging agents.	[8,20,36,37,38,39]
Temperature Beverages and Esophageal Cancer Risk	Not CAF itself, but consumption of hot caffeinated beverages (>65 °C) is associated with an increased risk of esophageal squamous cell carcinoma (ESCC), likely due to thermal injury to the esophageal mucosa leading to chronic inflammation and carcinogenesis.	[40,41,42,43,44]

**Table 2:** Summary of caffeine’s effects across various domains.

Area of Interest	Effect of Caffeine	Cited Studies
Psychiatric Disorders: Depression	Reduces depressive symptoms in moderate amounts; timing of intake affects depression risk; excessive intake and withdrawal are linked to increased depressive tendencies.	[45,46,47,48,49,50,51,52]
Psychiatric Disorders	Increases risk of anxiety, especially at higher doses; dose-dependent effects noted across populations, including adolescents.	[53,54]
Parkinson’s Disease	Reduces risk; improves motor functions and cognitive outcomes.	[55,56,57,58,59,60]
Migraine and Headaches	Enhances the effect of analgesics; beneficial in moderate amounts; excessive consumption or acute withdrawal can trigger or exacerbate migraines.	[61,62,63,64,65,66,67,68]
Stroke	Reduces stroke risk in certain populations; potential diagnostic value based on CAF metabolites.	[69,70,71,72]
Cognitive Performance and Dementia	Enhances alertness, attention, and memory; improves reaction time in certain contexts; may impair working memory due to increased metabolic demand; reduces prevalence and risk of dementia and Alzheimer’s; has neuroprotective effects due to anti-inflammatory and anti-oxidant properties.	[73,74,75,76,77,78,79,80,81,82,83,84,85,86,87,88,89,90,91]
Long-Term Caffeine Consumption	Associated with potential for tolerance and withdrawal symptoms but lacks classical addiction profile; ergogenic effects vary with habitual intake and dosing.	[92,93,94,95,96,97,98]
Limitations and Risks of Therapeutic Caffeine Use	Effective anticancer doses of caffeine in vitro exceed safe levels in humans. High doses may cause arrhythmias, seizures, or toxicity, especially in slow metabolizers. Metabolic variability, scarce clinical data, and side effects limit its use.	[1,99]

## Abbreviations

The following abbreviations are used in this manuscript:

A2AR	Adenosine A2A receptor
AD	Alzheimer’s disease
ADORA2A	Adenosine A2A receptor gene
ALS	Amyotrophic lateral sclerosis
ATM/ATR	Ataxia Telangiectasia Mutated/Ataxia Telangiectasia and Rad3-related kinases
ATR-Chk1	Ataxia Telangiectasia and Rad3-related kinase–Checkpoint kinase 1 (DNA damage response pathway)
BRCA1	Breast cancer gene 1
CAF	Caffeine (1,3,7-trimethylxanthine)
cAMP	Cyclic adenosine monophosphate
CI	Confidence interval
CUD	Caffeine use disorder
CYP1A2	Cytochrome P450 1A2 (liver enzyme metabolizing caffeine)
DASS	Depression, Anxiety, Stress Scale
EAC	Esophageal adenocarcinoma
EEG	Electroencephalography
ESCC	Esophageal squamous cell carcinoma
ESRD	End-stage renal disease
FDA	Food and Drug Administration
G2/M	G2/M phase cell cycle checkpoint
G6PD	Glucose-6-phosphate dehydrogenase
HIT-6	Headache Impact Test-6
HPA axis	Hypothalamic–pituitary–adrenal axis
MCI	Mild cognitive impairment
MDR	Multidrug resistance
NHANES	National Health and Nutrition Examination Survey
NK cell	Natural killer cell
OR	Odds ratio
PLGA	Poly(lactic-co-glycolic acid)
ROS	Reactive oxygen species
SIRT3	Sirtuin 3 (mitochondrial deacetylase)
SNP	Single-nucleotide polymorphism
SOD2	Superoxide dismutase 2
Th1	T-helper type 1 lymphocyte
TSD	Total sleep deprivation
UPDRS III	Unified Parkinson’s Disease Rating Scale, Part III (motor examination)
WM	Working memory
XO	Xanthine oxidase
